# Benign non-immune cells in tumor microenvironment

**DOI:** 10.3389/fimmu.2025.1561577

**Published:** 2025-04-03

**Authors:** Shaowen Liu, Chunhui Liu, Yuan He, Jun Li

**Affiliations:** ^1^ State Key Laboratory of Natural Medicines, School of Basic Medicine and Clinical Pharmacy, China Pharmaceutical University, Nanjing, China; ^2^ The First Affiliated Hospital, College of Clinical Medicine of Henan University of Science and Technology, Luoyang, China; ^3^ Henan Key Laboratory of Molecular Pathology, Zhengzhou, China; ^4^ Department of Molecular Pathology, Affiliated Cancer Hospital of Zhengzhou University and Henan Cancer Hospital, Zhengzhou, China

**Keywords:** tumor microenvironment, benign non-immune cells, cancer-associated fibroblasts, endothelial cells, pericytes, adipocytes, schwann cells, tumor heterogeneity

## Abstract

The tumor microenvironment (TME) is a highly complex and continuous evolving ecosystem, consisting of a diverse array of cellular and non-cellular components. Among these, benign non-immune cells, including cancer-associated fibroblasts (CAFs), adipocytes, endothelial cells (ECs), pericytes (PCs), Schwann cells (SCs) and others, are crucial factors for tumor development. Benign non-immune cells within the TME interact with both tumor cells and immune cells. These interactions contribute to tumor progression through both direct contact and indirect communication. Numerous studies have highlighted the role that benign non-immune cells exert on tumor progression and potential tumor-promoting mechanisms via multiple signaling pathways and factors. However, these benign non-immune cells may play different roles across cancer types. Therefore, it is important to understand the potential roles of benign non-immune cells within the TME based on tumor heterogeneity. A deep understanding allows us to develop novel cancer therapies by targeting these cells. In this review, we will introduce several types of benign non-immune cells that exert on different cancer types according to tumor heterogeneity and their roles in the TME.

## 1 Introduction

Research in neoplasm biology have traditionally focused on the proliferation and suppression of primary tumors. However, emerging studies have highlighted the indispensable role of benign non-immune cells surrounding cancer cells. Primary cancer cells exhibit increased heterogeneity when exposed to diverse stressful environment, such as hypoxia and acidification. This heterogeneity, in turn, contributes to the changes in the surrounding tumor microenvironment (TME) which subsequently promotes tumor progression and enhances aggressiveness (1).

Solid tumors are highly complex tissues composed of cancer cells that exhibit considerable heterogeneity in composition and evolutionary stages. Various components in TME, such as stromal cells, extracellular matrix (ECM), immune cells, blood/lymphatic vessels and nerve terminals, continuously and actively reshape the local immune response, through diverse signal molecules (2). The TME of most solid tumors is characterized by a markedly immunosuppressive milieu, including hypoxia, acidification, metabolic dysregulation, immune evasion and aberrant angiogenesis (3, 4). The chemical properties of hypoxia and acidification effectively inhibit the activation of immune cells. Hypoxia is a hallmark of most solid tumors and a defining feature of the TME that often leads to benign non-immune cells dysregulation, thus facilitating tumor growth (5). As tumors grow rapidly, an inadequate blood supply leads to hypoxia, especially in the tumor core (6). Hypoxia, along with the overexpression of hypoxia-inducible factors 1 and 2 alpha (HIF-1α and HIF-2α), which are key mediators of the tumor response within the TME (7). Under hypoxic condition, the activation of HIFs and their downstream signaling cascades, such as C-X-C chemokine receptor (CXCR)4, macrophage colony-stimulating factor receptor (M-CSFR) and CD47, modulates tumor-specific immune responses by inducing the production of immunosuppressive cytokines and growth factors, promoting tumor immune evasion and ultimately stimulating tumorigenesis (8, 9).

In addition, it is well established that TME is characterized by a low pH (3). Both tumor cells and immune cells, with elevated glycolytic activity, contribute to the accumulation of lactic acid both intercellularly and extracellularly. This accumulation results in a low intratumor pH and high extracellular lactic acid (10). This acid microenvironment may be associated with enhanced tumor local invasion and chemotherapy resistance (11, 12). Furthermore, tumor cells are surrounded by a physical barrier composed of stromal cells and the ECM (13). This barrier is closely associated with cancer associated fibroblast (CAFs) that secret a host number of fibers, contributing to a rigid and dense ECM structure (14), thereby hindering the infiltration of immune cells into the tumor and limiting their ability to eliminate cancer cells.

Hematological neoplasms refer to tumors that originate within hematopoietic tissue, such as the bone marrow and immune cells (15). These malignancies can originate from either the clonal evolution of hemopoietic stem cells (HSC) or the differentiation of progenitors with immune potential (16). Unlike solid tumors, hematological malignancies exist within a distinct tissue microenvironment, known as the bone marrow microenvironment (BMME) or bone marrow niche. This niche consists of HSCs, secondary lymphoid organs and the bone marrow composition. Similar to solid tumors, hematological tumors consist of cellular and non-cellular components. The cellular components include hematopoietic cells and non-hematopoietic cells, while the non-cellular components consist of ECM protein and various soluble factors such as cytokines, chemokines, growth factors, *etc*. (17).

The TME of hemopoietic malignancies is crucial for its progression. Various cell types within the proliferative niche of lymph nodes, bone marrow, and secondary lymphoid organs produce growth factors that support tumor survival. Non-hematopoietic stromal cells, the ECM, lymphocytes, and myeloid cells, within the TME, undergo functional and phenotypic changes. For example, macrophages within the TME can differentiate into tumor associated macrophages (TAMs) to promote tumor growth and immunosuppression. The dynamic interplay between hematopoietic tumor cells and the TME actively fosters a tumor-permissive niche, profoundly influences blood cancer progression by enabling immune evasion mechanisms and shaping the subsequent treatment response. Furthermore, alterations in metabolite availability within the TME, may disrupt immune homeostasis and impair the functions of microenvironment cells. Consequently, the essential function of this niche in maintaining homeostasis in bone marrow and secondary lymphoid organs is subverted to support cancer development (18–22). Studies have demonstrated that leukemia cells hijack the BM niche, remodeling it to create a microenvironment conducive to their survival. As a result, leukemia cells transform into leukemic stem cells, which grow more quickly than the hematopoietic cells, by utilizing the same mechanism as the hematopoietic stem cells (23, 24).

However, the TME varies according to tumor types, which makes it necessary for us to treat cancers based on tumor heterogeneity. This heterogeneity may derive from many aspects. For instance, pancreatic ductal adenocarcinoma (PDAC) is characterized by secreting large amounts of hyaluronan and an abundant deposition of ECM components, resulting in a dense matrix and hypovascular nature. This structure significantly reduces the delivery of chemotherapy drugs (25, 26). Hepatocellular carcinoma (HCC) is distinguished by its highly vascularized nature. HCC cells are capable of producing high amounts of vascular endothelial growth factor (VEGF) and angiopoietin (Ang)2, which stimulate the proliferation and migration of endothelial cells (ECs), leading to a highly vascular nature (27–29). Breast tissue is rich in fat, which leads to the occurrence of more adipocytes in breast cancer (BC) compared to other cancers. These adipocytes are closely related to the progression of BC (30). Besides, tumors can be classified into hot tumors and cold tumors based on the presence and arrangement of immune cells within the TME (31). For example, some patients with melanoma exhibit characteristics of hot tumors, with high levels of immune infiltration within the TME (32). Pancreatic cancer and glioblastoma are often defined as cold tumors due to the low infiltration of immune cells (33, 34). Genetic mutations also confer the difference in the TME across cancer types. These mutations often lead to alterations in the signaling cascades in the TME. KRAS mutation frequently occurs in pancreatic cancer, colorectal cancer (CRC) and non-small cell lung cancer (NSCLC) and this mutation significantly restricts T cell infiltration, recruiting suppressive immune cells into the TME (35). Therefore, understanding the heterogeneity is critical to cancer therapy. As pancreatic cancer is characterized by dense matrix induced by hyaluronan, strategies to eliminate hyaluronan to promote drug infiltration shed light on pancreatic cancer treatment. Moreover, methods that transform cold tumors into hot tumors may effectively enhance the therapeutic effect of immune checkpoint inhibitors (ICI).

TME is a highly complicated and dynamic system and the components of TME may vary across cancer types. Thus, it is important to understand the mechanisms that these benign non-immune cells contribute to cancer progression based on cancer types.

### 1.1 TME: History and definition

The concept of TME has undergone significant evolution over time. In 1863, Rudolph Virchow first documented the interaction between tumor cells and their microenvironment, and he reported the leukocyte infiltration within the TME (36). In 1889, Stephen Paget, proposed the ‘soil and seed’ hypothesis, suggesting that tumor progression is intimately linked to cellular microenvironment (37). In 1993, Ioannides and Whiteside formally introduced the term ‘TME’ (38), and they thought that the TME not only includes the structure, function and metabolism of the tumor tissue, but also is related to the internal environment of the tumor cell itself.

Non-malignant cells within the TME, including immune cells and benign non-immune cells, have been the subject of extensive research. CAFs, as the most important components in the TME, have been studied for a long time. In 1858, Virchow first identified spindle-like cells capable of secreting collagen. In cancers, hyperactivated fibroblasts are referred to as CAFs (39). An early study showed that BC stroma-derived fibroblasts exhibit a strikingly different morphology and growth properties compared with normal fibroblasts (40). They co-cultured normal fibroblasts and Hela cells, proving that fibroblasts showed a cytotoxic effect on tumor cells (41). However, subsequent research revealed that CAFs could also support prostatic tumor growth both *in vitro* and *in vivo* (42, 43).

The history of immune cells dates back to 1970s (42), Eva Klein was among the first to highlight the presence of tumor-attacking cells at tumor cites (44). In the 1980s, Rosenberg reported that adoptive transfer of tumor infiltrating lymphocytes (TILs) effectively inhibited tumor progression (45). Numerous studies have also documented macrophage infiltration in tumors (46, 47). While early investigations suggested that TAMs might suppress tumor growth, however, more recent findings indicated that TAMs promote tumor progression instead (48–50). Myeloid-derived suppressor cells (MDSCs) were initially described as veto cells, null cells, or natural inhibitory (NS) cells (51). In the mid-1960s, it was reported that NS cells could induce a leukemoid reaction in the tumor-bearing mouse, which was associated with the duration of mammary carcinoma or A280 tumor growth and myeloid-cell infiltration (52, 53). Later these cells were formally defined as MDSCs (54), which play a critical role in immunosuppression within the TME.

TME is a highly structural ecosystem comprising a diverse array of cellular and non-cellular components. Non-cellular components include ECM, tumor vasculature, chemokines and a variety of soluble factors secreted by different cells. The cellular components consist of versatile types of cells, including immune cells, stromal cells, CAFs, ECs and others (55). These components make TME a dynamic and complex three-dimensional (3D) environment. Particularly benign non-immune cells, which are crucial parts of the TME, contribute to the tumorigenesis by secreting cytokines and recruiting immunosuppressive cells (56). For example, tumor associated stromal cells(TASC) can communicate with other types in the TME through cell-cell interactions or paracrine signaling, releasing cytokines and mediators to promote the tumor progression (57). Additionally, emerging mechanisms of cell interactions, such as the release of exosomes, digestion of cell-free DNA and the clearance of apoptotic bodies (58), also play tumor-promoting or tumor-inhibiting roles depending on tumor heterogeneity. These findings suggest that targeting benign non-immune cells within the TME represents a promising strategy to suppress tumor development and improve cancer prognosis.

Immune cells within the TME are often affected by the microenvironment to exhibit immunosuppressive effect. However, extensive evidence highlights the close association between benign non-immune cells within the TME and tumor progression, targeting these cells provides a novel pathway for treating cancers. For example, fibroblast activation protein (FAP), highly expressed in CAFs across various cancer types but minimally expressed under normal conditions (59), has emerged as a promising target. Numerous studies have explored FAP-targeted therapies and yield encouraging outcomes that underscore the potential of focusing on benign non-immune cells in the TME to combat cancer (60–62).

## 2 Benign non-immune cell types

**Figure 1:**
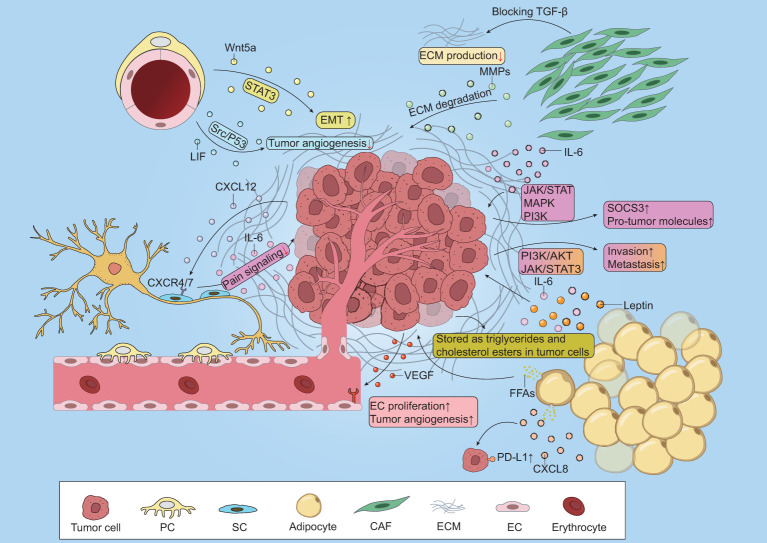
Crosstalk between benign non-immune cells and cancer cells. CAFs secret TGF-β to promote ECM deposition and blocking TGF-β effectively inhibits this procession. Besides, CAFs-derived MMPS contribute to ECM degradation. IL-6 secreted from CAFs promotes the release of SOCS3 and various pro-tumor molecules via JAK/STAT, MAPK and PI3K pathway. Tumor-derived VEGF promotes EC proliferation and neovascularization. Adipocytes release FFAs to supply energy for tumor survival and stored as triglycerides and cholesterol esters in tumor cells. Moreover, adipocytes secret IL-6 and leptin to enhance tumor invasion and metastasis via PI3K/AKT and JAK/STAT3 pathway. CXCL8-derived from adipocytes upregulates PD-L1 expression on tumors. Tumor cells release CXCL12 to attract SCs to the tumor cite by binding to CXCR4/7 and SCs produce IL-6, collectively weakening pain signaling. Overproduced Wnt5a promotes the EMT of cancer cells via STAT3 signaling pathway. PCs-derived LIF inhibits tumor angiogenesis via Src/P53 signaling pathway.

Stromal cells are essential cellular components in the TME that participate in tumor immune response, metabolism, invasion, drug resistance, *etc*. Stromal cells can be classified into multiple subtypes based on different criteria, with the primary subtypes being CAFs, ECs, pericytes (PCs) and adipocytes (57). Recent studies also highlighted that Schwann cells (SCs) within the TME may be associated with tumor progression (**Figure 1**).

These cells play different roles in the TME, collectively contribute to tumor progression (63). For example, CAFs are benign non-immune cells that have been extensively studied. CAFs within the TME are capable of secreting a wide array of factors to inhibit immune response and remodeling the ECM to promote cancer metastasis or inhibit drug infiltration (64). Adipocytes serve as an important energy source for tumor survival and secret adipokines to impact cancer progression (65). These benign non-immune cells play a pivotal role in tumor progression and the role may vary across diverse cancer types, thus understanding the heterogeneity of benign non-immune cells is crucial for cancer therapy.

### 2.1 Cancer-associated fibroblasts

Fibroblasts are quiescent cells that participate in sustaining the homeostasis in connective tissues by producing connective ECM and secreting various cytokines, such as transforming growth factor-β (TGF-β) (66). However, fibroblasts are often persistently activated to participate in tissue repair, resulting in tissue fibrosis in the context of cancer or persistent inflammatory stimulation (67). The persistent wound healing-like process in the TME leads to the development of a particularly active subset of fibroblasts (64), termed CAFs. Compared with normal fibroblasts, CAFs are endowed with higher metabolism, increased proliferation and migration capacities, such as in BC, PDAC and CRC (68–70). In addition, CAFs differ from normal fibroblasts in their cellular morphology. While mature fibroblasts display a thin, wavy and compact spindle-like shape. CAFs, often classified as immature fibroblasts, have a larger, rounder spindle-shape with conspicuous nucleoli (57).

CAFs are known to secret diverse cytokines and remodel ECM. CAFs can secret multiple cytokines, chemokines, including interleukin (IL)-6, IL-8, IL-10, IL-4, tumor necrosis factor (TNF), TGF-β, C-X-C motif chemokine ligand (CXCL)9, CXCL10, C-C motif chemokine ligand (CCL)5, CCL2, *etc*., that may directly or indirectly contribute to tumor progression (64). The remodeling of ECM is a complicated process, including the activation of a wide array of signaling pathways and participation of transcription factors (71). CAFs are capable of synthesizing various proteins, including multiple types of collagens (I, III, IV, V), laminins, fibronectins and hyaluronan that constitute the ECM (72, 73). Lysyl oxidase (LOX) or lysyl hydroxylase 2 helps to promote the cross-link of collagens to enhance ECM stiffness (74, 75). Besides, TGF-β plays a crucial role in the ECM remodeling mediated by CAFs. For example, TGF-β stimulates CAFs to synthesize collagen, thereby facilitating the ECM deposition (76). Also, under the effect of TGF-β, CAFs are capable of producing hyaluronan that further increases ECM deposition and enhances its stiffness (77). In gastric cancer, TGF-β activates downstream Smad2/3 to upregulate the hyaluronan and proteoglycan link protein 1 (HAPLN1) production, which drives ECM remodeling (78). CAFs also remodel the ECM under the effect of heat shock factor-1 (HSF-1). Mechanically, Dickkopf-3 (DKK3), highly expressed in BC, CRC and ovarian cancer, is upregulated under the effect of HSF-1. This upregulation enhances the activation of classical Wnt signaling, leading to the reduced degradation of Yes-associated protein (YAP) and transcriptional coactivator with PDZ-binding motif (TAZ), this, in turn, results in ECM remodeling mediated by CAFs (79). However, the formation of ECM is a dynamic process, CAFs produce matrix metalloproteinases (MMPs) to promote the degradation of ECM, leading to enhanced motility and invasion of cancer cells (64, 80). In BC, TGF-β induces significant expression of MMP2 and MMP9 in a Smad3/4-depend manner. Overexpressed MMP2 and MMP9 degrade ECM to promote BC metastasis (81). Moreover, MMPs may serve as crucial components of the angiogenic switch. CAFs-derived MMPs degrade the ECM that allows VEGFA to interact with vascular endothelial growth factor receptor (VEGFR), thus promoting tumor angiogenesis (82).

Beyond the complexity of CAFs modulating the mechanisms of cancer progression. CAFs also exhibit high heterogeneity in their origin and phenotype, such heterogeneity is strikingly associated with tumor progression (83). Three subtypes of CAFs have been identified across multiple cancers: inflammatory cancer associated fibroblasts (iCAFs), matrix-remodeling myofibroblastic cancer associated fibroblasts (myCAFs) and antigen-presenting cancer associated fibroblasts (apCAFs) with histocompatibility complex class II (MHC-II) expression (84). iCAFs, which have low α-smooth muscle actin (αSMA) expression, are found scattered throughout the tumor mesenchyme and often located near to the blood vessels, producing high amounts of inflammatory cytokines, such as IL-6. In contrast, myCAFs are located in the periglandular region and directly interact with tumor cells, exhibiting high αSMA expression (85, 86). Besides, myCAFs are characterized by high expression of TGF-β signaling and leucin-rich repeat containing 15 (LRRC15) (84). Recent studies have also identified additional subtypes of CAFs in several carcinoma types, such as lipid-laden CAFs, a subtype of CAFs with adipocyte associated gene expression identified in CRC and pancreatic cancer (87, 88). Moreover, a new subtype of CAFs with highly activated metabolic state, termed metabolic cancer associated fibroblasts (meCAFs), were identified in loose type PDAC. meCAFs are marked by enhanced glycolytic activity, while the associated cancer cells predominantly rely on oxidative phosphorylation as their metabolic pathway, rather than glycolysis (89). Furthermore, extracellular matrix cancer associated fibroblasts (eCAFs) were identified in gastric cancer (90). Therefore, the classification of CAFs exhibits high heterogeneity across different tumor types and the roles that they exert on tumor cells may not exactly the same.

One particular sample is the role of CAFs in PDAC, primarily focusing on the subtypes iCAFs and myCAFs (91). iCAFs are known to influence tumor progression through multiple mechanisms. One key mechanism is the upregulation of IL-6 expression in iCAFs, which contributes to PDAC development by fostering an immunosuppressive microenvironment (91). IL-6 activates several signaling pathways, including JAK/STAT, MAPK and PI3K pathway, leading to the release of negative regulator suppressor of cytokine signaling 3 (SOCS3) and various pro-tumor molecules (92). Preclinical studies have shown that blocking IL-6 receptor enhances the therapeutic effect of programmed cell death ligand 1 (PD-L1) and inhibiting IL-6 expression exhibited promise in suppressing pancreatic cancer progression (93–95). Besides, CAFs can interact with cancer cells within the TME to induce the production of iCAF phenotype. iCAFs are characterized by hypoxia-associated gene expression profile and metabolic features that are rich in the PDAC. Tumor-derived cytokines (eg. IL-6) may transform normal fibroblasts (NFs) into iCAF, thereby promoting the pancreatic cancer progression in a HIF-1α dependent way (96). Moreover, cirCUL2 is specifically expressed in CAFs, and the enrichment of cirCUL2 in PDAC can also induce the transformation of NFs into iCAFs and drive PDAC progression by overexpressing IL-6 (97). myCAFs, on the other hand, have been reported to participate in pancreatic cancer progression. A study co-cultured naive pancreatic stellate cells (PSC), a precursor population of CAFs, and KrasLSL-G12D/+; Trp53LSL-R172H/+; Pdx1-Cre (KPC) mice derived organoids that demonstrating TGF-β signaling can promote the transformation of CAFs into myCAFs while inhibiting iCAFs formation by downregulating interleukin-1 receptor 1 (IL1R1) expression in CAFs (98). Blocking TGF-β can disrupt the myCAFs associated barrier and enhance the infiltration of drugs into the pancreatic cancer (99). Targeting transforming growth factor beta receptor 1 (TGFβR1) in combination with gemcitabine increases drug perfusion into the tumor core and improves the therapeutic efficacy in pancreatic cancer (100). Furthermore, myCAFs can promote PDAC progression via SOX4/MMP11 signaling axis. Recently, apCAFs were identified in the pancreatic cancer, but not as abundant as iCAFs and myCAFs (91, 101). Though apCAFs sustain low levels in pancreatic cancer, their expression of MHCII molecules enables them to present antigenic peptides and participate in tumor immune regulation (84).

At least four distinct CAFs’ subtypes have been identified in human BC, each with unique properties and characteristics: CAF-S1, CAF-S2, CAF-S3, CAF-S4 (102). CAF-S1 and CAF-S4 are myofibroblasts that accumulate in aggressive BC, whereas CAF-S2 and CAF-S3 are present both in tumor cells and normal cells (102), implicating that they may serve as NFs in the TME. CAF-S1 attracts CD4^+^CD25^+^ T lymphocytes by secreting CXCL12 and sustain their presence through OX40 ligand (OX40L) and junctional adhesion molecule 2 (JAM2). CAF-S1 supports T lymphocytes survival and transforms them into CD25^High^FOXP3^High^ phenotype, namely regulatory T cells (Tregs), through the B7 homolog 3 (B7H3), CD73, and dipeptidyl peptidase 4 (DPP4). Additionally, CAF-S1 enhances the efficacy of these cells to further impair T cell proliferation (102). In the meanwhile, CAF-S1 is capable of promoting luminal BC metastasis through cadherin 11 (CDH11)/osteoblast cadherin (103). The infiltration of Tregs into the BC stroma is associated with CD73 expression in CAF-S1 and the blockade of CD73 can effectively reduce CD73 mediated immunosuppression (104). These findings suggest that CD73 could serve as a promising therapeutic target to overcome the immunosuppressive microenvironment induced by CAF-S1. In contrast, CAF-S4 exhibits a negative correlation with Tregs’ infiltration, suggesting that CAF-S4 may exert positive anti-tumor effect in the TME of BC.

Similarly, CAFs are also crucial components in the progression of hepatic pathogenesis. Single-cell RNA sequencing has classified CAFs subpopulation in liver cancer into iCAFs and myCAFs (105). The hyaluronan synthase 2 (HAS2) is strikingly upregulated in myCAFs and has been demonstrated to promote cholangiocarcinoma (ICC) progression. iCAFs can directly interact with tumor cells through secreting hepatocyte growth factor (HGF), thereby enhancing ICC growth (105). Additionally, some new subtypes of CAFs have been identified in HCC, namely vascular cancer associated fibroblasts (vCAFs), mesenchymal cancer associated fibroblasts (mCAFs), lipid processing cancer associated fibroblasts (lpCAFs), apCAFs and CD36^+^ CAFs. Among these, CD36^+^ CAFs are particularly notable for promoting HCC progression. CD36 facilitates the uptake of oxidized low-density lipoprotein (LDL), which induces macrophage migration inhibitory factor (MIF) expression in CD36^+^ CAFs via a lipid peroxidation/p38/CEBPβ signaling pathway. Consequently, CD33^+^ MDSCs, which are associated with immunosuppressive microenvironment, are recruited into the HCC tissue in a manner dependent on both macrophage MIF and CD74 (106). CD36 may serve as a potential target for tumor treatment, and combining CD36 inhibitors with anti-programmed death protein 1 (PD-1) has been shown to restore the anti-tumor immune response of T cells (106). A recent study also found a new subpopulation of CAFs in liver cancer, termed F5-CAF, which is associated with increased cancer stemness and poor prognosis. Although the exact tumor-promoting mechanisms remain unclear, F5-CAF may facilitate liver cancer progression by enhancing the stemness of tumor cells (107).

The study of CAFs’ subpopulation in lung cancer is not as clear as that studied in pancreatic cancer. CAFs in NSCLC can be divided into three subtypes based on their function in the TME, termed subtype-1, subtype-2 and subtype-3. Subtype-1 plays a robust protective role of cancer cells. Subtype-2 provides moderate tumor protection effect, while subtype-3 plays a minimal protective role (108). Both subtype-1 and subtype-2 highly express HGF and fibroblast growth factor (FGF)7, which supports NSCLC cell survival through inhibiting anaplastic lymphoma kinase (ALK) inhibitors and epidermal growth factor receptor (EGFR) inhibitors. In contrast, subtype-3 is often associated with good prognosis, enhancing T lymphocytes and monocytes infiltration via a wide array of chemokines, such as CXCL11, CXCL12 and CXCL14 (108). For cases with subtype-1or -2 CAFs, targeting HGF and FGF7 may hold promise for improving lung cancer therapy. But TME is a complicated system, including the participation of various signaling pathways, whether targeting these factors has an influence on other pathways that are associated with tumor progression needs further exploration. Another study divided CAFs into seven subtypes in lung cancer: clusters 1 to clusters 7 (109). These subtypes likely influence tumor progression through various mechanisms. For example, cluster-1 is associated with epithelial-mesenchymal transition (EMT) and is characterized by high expression of ECM proteins and TGF-β. TGF-β is often thought to promote tumor growth by enhancing glycolysis and lactic acid production in CAFs, providing metabolic substrates for tumor cells (110). This suggests that cluster-1 is probably a tumor-promoting phenotype of CAFs in lung cancer.

CAFs, as one of the most studied benign non-immune cells in the TME, play a diverse role in different cancers and exhibit a significant heterogeneity. Strategies to target CAFs hold promise to cancer therapy. There are already many methods to treat cancer based on CAFs, including blocking TGF-β mediated signaling, targeting FAP, *etc*. Some of these methods have already moved into clinical research. A study used galunisertib, a TGF-β receptor inhibitor, plus gemcitabine effectively improve the overall survival rate of patients with pancreatic cancer compared to gemcitabine alone. And this combination only added minimal toxicity (111). Additionally, galunisertibl plus neoadjuvant chemotherapy allowed most patients with locally advanced rectal cancer to garner better complete response rate (112). It is reported that a dual-target agonist aimed at FAP and 4-1BB induced effective drug response in patients with advanced solid tumor without the occurrence of liver toxicity (113). These clinical trials revealed that targeting CAFs within the TME is promising for cancer therapy. However, there are potential risks behind these strategies. For instance, the blocking of TGF-β may lead to unexpected adverse effect. An inhibitor of TGF-β1 receptor, was reported to induce anemia, fatigue, hypoalbuminemia, *etc*. (114). The potential mechanism can be attributed to the autoimmune response. Moreover, it is of great significance to combine TGF-β with other therapies, such as chemotherapy drugs and ICI. Single drug therapy may not acquire persistent therapeutic effect and the drug tolerance often occurred. As for FAP targeted therapy, the expression of FAP in normal tissue may result in unexpected toxicity in normal tissues or organs and it would benefit from muti-target and drug combination.

### 2.2 Endothelial cells

The formation of tumor blood vessels is crucial for tumor growth and metastasis, supplying the necessary energy and nutrients to sustain tumor progression (115). ECs, as indispensable components of the vascular system, construct the inner lining of all sub-vascular compartments, ensuring the delivery of nutrients and energy to distant tissues and organs (116). Under normal physiological condition, ECs remain in a quiescent state, but can be activated into tumor-associated endothelial cells (TECs) through multiple mechanisms. For example, the TME is often characterized by hypoxia, which upregulates VEGF release in a paracrine manner, stimulating EC proliferation and blood vessel formation (117). The regulation of ECs is closely related to tumor angiogenesis in the context of cancer or tumor. VEGF/VEGFR is a classical signaling pathway that promotes EC proliferation. There are also other pathways that may contribute to EC proliferation. Ang-Tie axis on ECs helps to regulate blood vessel formation, stability and inflammation (118). In normal condition, Ang1 binds to Tie2 and activates Tie2 through phosphorylating Tie2, which activates downstream signals to regulate vascular stability. However, Ang1 and Ang2 may play opposite roles in blood vessel formation in the context of cancer. Ang2, low in the normal physiological condition but often highly expressed in the context of cancer, competitively binds to the Tie2 receptor with Ang1, reducing the stability of blood vessels and establishing a vascular environment that facilitates cancer cell metastasis (118). Notch signaling pathway is also actively involved in the process of tumor angiogenesis. ECs express delta-like protein 4 (DLL4) and Notch 1 when stimulated by VEGF. The DLL4/Notch transduction is able to negatively reduce the sensitivity of ECs to VEGF, thus suppressing EC proliferation and sprouting. At the same time, DLL4 reduces VEGFR2 expression in ECs to further inhibit tumor angiogenesis (119). In addition to their role in vascular formation, ECs interact closely with immune cells due to their location on the interior surface of blood vessels, influencing the tumor immune therapy. For example, TECs highly express natural killer group 2D (NKG2D) ligands, inducing nature killer (NK)-intrinsic signal that desensitizes the immune response of NK cells, weakening pro-inflammatory cytokines and cytotoxic granule mediated tumor-killing effects (120). Targeting ECs has emerged as one of the most promising strategies for cancer adjuvant therapy. However, the heterogeneity of ECs across different cancer types necessitates a deeper understanding of their roles in diverse tumoral contexts. Elucidating the specific functions of ECs in various cancers is essential for developing more effective therapeutic approaches.

HCC is often associated with poor prognosis partly due to its highly vascularized nature. HCC cells can produce significant amounts of VEGF, ECs specific vasculogenic and angiogenic growth factors, contributing to the angiogenesis of many solid tumors. Tumor-derived VEGF activates quiescent ECs in a paracrine manner, thereby driving ECs’ proliferation and HCC metastasis (27–29). However, researchers have discovered additional mechanisms through which ECs contribute to HCC invasion and metastasis. Diacylglycerol kinase gamma (DGKG), specifically hyper-expressed in tumor vascular ECs of HCC, promotes tumor angiogenesis and facilitates the formation of an immunosuppressive microenvironment under hypoxia (121). Mechanically, HIF-α upregulates DGKG expression and recruits ubiquitin-specific peptidase 16 to stabilize ZEB2 through deubiquitination, enhancing TGF-β1 secretion. This cascade promotes blood vessel formation and Treg differentiation, forming an immunosuppressive microenvironment to further support HCC survival (121). Besides, ECs in HCC exhibit increased activity in the fructose metabolism pathway, notably through the upregulation of fructose transporter SLC2A5 and the fructose-metabolizing enzyme ketohexokinase (KHK). Hypoxia-induced elevated fructose metabolism, activating AMPK to fuel mitochondrial respiration and enhance EC migration (122). Targeting this pathway may present a promising strategy to inhibit HCC angiogenesis. Additionally, sphingosine-1-phosphate receptor 1 (S1PR1), which is specifically and highly expressed in HCC compared to para-tumor tissues, has been shown to promote HCC angiogenesis by reducing ceramide levels (123). This suggests that S1PR1 may be a new target to liver cancer therapy. ECs can also influence the function of immune cells in the TME. A recent study discovered a novel subtype of CXCL12^+^ TECs that promote the immune resistance of HCC. These cells secret CXCL12, blocking the differentiation of CD8^+^ naive T cells to CD8^+^ cytotoxic T cells. CXCL12^+^ TECs also recruit MDSCs to the TME, establishing an immunosuppressive environment that impairs immune responses against HCC (124). Moreover, another study exhibited that TECs induce the exhaustion of tumor-infiltrating CD8^+^ T cells in HCC by expressing glycoprotein nonmetastatic melanoma protein B (GPNMB). Silencing GPNMB largely reversed this immunosuppressive effect (125).

Lymph nodes metastasis is an important hallmark of gastric cancer progression. A co-culture experiment with gastric cancer cells and lymphatic endothelial cells (LECs) demonstrated that cancer cells can stimulate LECs to secret CXCL1 via NF-κB pathway. CXCL1, in turn, promote LECs’ migration and tube formation, thereby facilitating gastric cancer metastasis (126). Similarly, CXCL1 secreted by tumor associated lymphatic endothelial cells (TLECs) contributes to gastric cancer adhesion, invasion and metastasis by activating integrin β1-FAK-AKT signaling pathway (127). These findings suggested that targeting CXCL1 may be a promising strategy to treat gastric cancer. Furthermore, gastric cancer cells overexpress Biglycan (BGN), which stimulates VEGF expression through the interaction between NF-κB and HIF-α. Overexpressed VEGF leads to gastric cancer angiogenesis in a chronic activation manner (128). Neuropilin-2, which is highly expressed in the tumor vessel lining has been shown to enhance VEGF-induced ECs’ proliferation and migration, highlighting its role in gastric cancer progression (129). Additional studies have identified other potential targets associated with gastric cancer metastasis. For instance, transglutaminase-2 (TGM2) and neuro oncological ventral antigen 2 (NOVA2) have been implicated in poor prognosis for gastric cancer patients (130, 131).

BC dissemination frequently involves in lymphatic vessels. Tumor-conditioned LECs promote BC angiogenesis and direct its dissemination and proliferation via CCL5. BC cells secret IL-6, which triggers STAT3 phosphorylation and downstream signal activation in LECs, thereby driving CCL5 expression in these cells (132). Besides, triple-negative breast cancer (TNBC) cells are capable of secreting plasminogen activator inhibitor-1 (PAI-1), which stimulates CCL5 secretion from LECs in a paracrine manner. CCL5, in turn, accelerates PAI-1 secretion, forming a positive loop that accelerates TNBC invasion and metastasis (133). Recently, a study proposed a novel mechanism through which ECs contribute to BC dispersion. Regulator of G-protein signaling 5 (RGS5)^+^ ECs can promote tumor lymph nodes dissemination and drug resistance by sensing oxidative stress (134), which provides a potential target for BC therapy.

ECs are also actively involved in CRC progression. The expression of G protein-coupled receptor 63 (GPR63) is significantly elevated in CRC. EC-derived sphingosine-1-phosphate (S1P) promotes CRC migration by enhancing the interaction between Src and GPR63, which subsequently initiates JAK2/STAT3 pathway activation (135). In CRC, adipocyte enhancer-binding protein 1 (AEBP1) is frequently upregulated in ECs. Knockdown of AEBP1 was proved to suppress tumorigenesis and micro-vessel formation. This has implications that AEBP1 may regulate genes that are closely associated with angiogenesis, including aquaporin 1 (AQP1) and periostin (POSTN) (136). Furthermore, CRC cells also actively interact with ECs within the TME. miR-221-3p is significantly upregulated in patients with CRC compared to healthy individuals. CRC derived extracellular vesicles (EVs) containing miR-221-3p regulate STAT3/VEGFR-2 signaling axis by targeting SOCS3, leading to enhanced EC proliferation, migration and angiogenesis (137). CRC-derived cationic amino acid transporter 1 (CAT-1) positive EVs, known for transporting extra arginine, facilitate ECs’ growth and blood vessel formation by upregulating arginine transport and cGMP metabolism (138). ECs are also convinced to enhance stemness of cancer cells when GLTSCR1 is knocked down. The depletion of GLTSCR1 promotes the transition of ECs into tip cells by regulating neuropilin-1 expression and upregulates JAG1 expression which is closely related to enhanced cancer cell stemness via activating notch signaling pathway (139).

ECs in the TME are often associated with tumor angiogenesis and dissemination. In addition to the classical angiogenesis signals, studies revealed other potential pro-angiogenic targets based on cancer types, such as DGKG in HCC, BGN in gastric cancer and AEBP1 in CRC. The emergence of these targets sheds light on cancer therapy. Besides, there are already many drugs gained good outcomes in the clinical practice. Bevacizumab, a VEGF receptor blocker, effectively slowed the progression of metastatic renal cancer (140). Bevacizumab in combination with atezolizumab (a type of PD-L1 inhibitor), exhibited encouraging outcome in patients with unresectable HCC (141). However, more than half of the subjects experienced grade 3 or 4 hypertension. Trebananib (a recombinant peptide-Fc fusion protein capable of disrupting the interaction between Ang1/2 and Tie2) may enhance the anti-tumor effect of Sunitinib (a VEGF receptor blocker) but as well as elevating the drug toxicity (142). Most of the drugs in the clinical practice based on muti-pathway blockade, such as VEGF, platelet derived growth factor (PDGF) and FGF. The blockade of a single pathway can trigger the compensatory effects in tumors, leading to drug resistance and increased tumor angiogenesis by activating other pro - angiogenic pathways (143, 144).

### 2.3 Pericytes

PCs are referred to as mural cells that envelop the capillaries, which actively interact with ECs to collectively regulate vascular formation, stabilization, remodeling and function (145). The common markers of PCs include chondroitin sulfate proteoglycan 4 (CSPG4), CD146 and platelet-derived growth factor receptor-beta (PDGFR-β) (146). As important components in the TME, PCs are involved in tumor progression, invasion and migration. Endothelium, an abundant source of platelet-derived growth factor B (PDGFB), is capable of recruiting PCs into the blood vessel by binding to PDGFR-β to sustain the integrity and stability of tumor blood vessels (147). Also, ECs produce nitric oxide (NO) to stimulate the release of VEGF from PCs, supporting EC proliferation and survival (148). This interaction between PDGFB and PDGFR-β also promotes PCs proliferation and migration by activating Ras superfamily proteins (147). However, high levels of PDGFB activate the PDGFR - β/cyclooxygenase-2 (COX-2) signaling pathway, increasing the levels of pro-inflammatory factors, thereby exacerbating the hematogenous metastasis of BC (149). Additionally, PCs also contribute to the formation of a pre-metastatic microenvironment. The ablation of krüppel-like factor 4 (KLF4) effectively suppresses PCs’ proliferation and lung metastasis (150). This suggests that PCs are endowed with high metastatic property in the context of cancer without knocking KLF4. Also, tumor-derived exosomes activate KLF4 in PCs, leading to enhanced ECM production and establishment of a fibronectin-rich niche which is conducive to the hematogenous metastasis (151). The metabolism of PCs may also undergo certain changes. Hexokinase 2(HK2)-driven glycolysis elevates in PCs and upregulates ROCK2-MLC2 mediated contractility. This contractility may lead to impaired blood vessel function (152). Additionally, PCs also interact with immune cells within the TME. PCs are capable of recruiting TAMs into the TME to form an immunosuppressive microenvironment by secreting large amounts of IL-33. Mechanically, PDGF-BB stimulates PCs and activates SOX7 transcriptional factor, leading to the release of IL-33 (153). PCs secret CXCL9 and CXCL12 to recruit CD8^+^ T cells expressing CXCR3 and CXCR4 into the TME. At the same time, CXCL12 promotes the expression of TGF-β and IL-10, which weakens the proliferation and antigen-presenting capacity of T cells (154). However, the function of PCs may vary across cancer types that it is better to understand the function of PCs based on cancer heterogeneity.

In addition to regulating the integrity and stability of the tumoral blood vessel. PCs may exert specific roles on CRC. PCs may serve as a potential promoter in the process of colorectal cancer liver metastasis (CRCLM). TRP channel-associated factor 2 (TCAF2), which serves as a partner protein of the transient receptor potential cation channel subfamily M member 8 (TRPM8), is overexpressed in PCs of CRCLM patients. TCAF2 inhibits TRPM8 expression and activation, leading to the secretion of Wnt5a. Overproduced Wnt5a is able to promote EMT via the activation of STAT3 signaling pathway (155). Furthermore, PCs secret high levels of TGF-β in response to the stimulation from CRC cells. TGF-β initiates the autocrine activation loop that produces insulin-like growth factor binding protein-3 (IGFBP-3) to promote cancer invasion and migration (156). Moreover, a study identified a novel subpopulation of PCs that highly express transcription factor 21 (TCF21) (157). The increased TCF21 is able to remodel the perivascular metastatic microenvironment which benefits from enhanced perivascular ECM stiffness, collagen rearrangement and basement membrane degradation (157). However, this pro-tumor effect can be reversed by increasing the production of integrin-α5, which activates FAK/PI3K/AKT/DNMT1 axis.

PCs exhibit a unique secretome, with high secretion of IL-32, in EGFR mutated lung cancer patients. The potential mechanism is that Yin-Yang 1 (YY1) signaling pathway upregulates the production of IL-32. IL-32 subsequently activates the β5-integrin-Src-Akt pathway to reduce the sensitivity to the third-generation tyrosine kinase inhibitors (TKIs) in EGFR mutated lung cancer patients (158). CD248 is expressed across tumor stromal cells, especially fibroblasts and PCs. However, the role that CD248^+^ PCs play on tumor is less studied than CD248^+^ fibroblasts. A recent study revealed that CD248 could suppress Wnt signaling and upregulates the expression of Osteopontin (OPN) and SERPINE1, which are associated with increased tumor volume. The upregulation of OPN and SERPINE1 collectively promotes tumor angiogenesis and supports lung cancer cell growth (159). HK2 positive PCs are characterized by elevated glycolysis, this elevation could upregulate ROCK2-MLC2 mediated contractility, resulting in aberrant blood vessel function, such as decreased blood vessel diameter and collagen, which are advantageous to lung cancer metastasis (152).

PCs are also involved in BC progression through multiple mechanisms. PDGFB is originally thought to sustain the stability and integrity of blood vessel. However, PDGFB-to-PDGFR-β tumor–stroma signaling also promotes the initiation and metastasis of cancer cells in the context of BC (160). Moreover, purified PCs are capable of supporting the rapid growth of BC cells when co-cultured with them. This supporting effect could be attributed to the establishment of a niche beneficial to BC proliferation (161). A recent study exhibited that PCs secret insulin-like growth factor 2 (IGF2), which plays a significant and specific pro- proliferative effect on BC. This pro- proliferative effect could be inhibited through suppressing IGF2-mediated signaling (162). Besides, extracellular nicotinamide phosphoribosyltransferase (eNAMPT), which is highly expressed in the TNBC, can promote the angiogenesis of TNBC by attracting and activating NG2^+^ PCs. This process can be achieved through synergizing with the classic factor PDGF-BB. It triggers the pro-inflammatory activation of PCs through the NF-κB signaling pathway, manifested by the overexpression of key chemokines (CXCL8, CXCL1, CCL2) and vascular cell adhesion molecule 1 (VCAM1) (163). However, PCs may also serve as potential inhibitors of BC progression. KAI1 (CD82), specific and highly expressed in PCs rather than ECs, is able to inhibit tumor angiogenesis through promoting the release of leukemia inhibitory factor (LIF) of PCs via Src/P53 pathway. In the meanwhile, KAI1 directly interacts with VEGF and PDGF to prevent binding to the receptors (164). This mechanism may provide us with a novel anti-angiogenic method.

PCs are crucial components that regulate the blood vessel stability. However, PCs are also involved in cancer progression by promoting cancer metastasis and angiogenesis. The study of PCs in multiple cancers provide us with some attractive targets. There are already drugs targeting PDGFR in the phase of clinical trial. However, single-target drugs do not acquire satisfying clinical outcomes. Combined therapy can synergistically exert anti-tumor effects, reduce the occurrence of adverse effect and drug tolerance. Olaratumab combined with doxorubicin prolongs the overall survival rate and reduces the occurrence of adverse events compared to single drug therapy of doxorubicin (165).

### 2.4 Adipocytes

Adipose tissue, an active metabolic store and endocrine organ capable of secreting a wide array of adipokines, plays a significant role in promoting tumor development (166). Adipocytes primarily store long-chain fatty acids as triacylglycerol and cholesterol esters in the form of lipid droplets (167). Adipose tissue can be divided into three types based on adipocytes constituents: brown adipose tissue, white adipose tissue and beige adipose tissue (168). White adipose tissue constitutes more than 95% of the total fat tissue, while brown adipose tissue accounts for 1-2%. Beige adipose tissue is more difficult to be quantified, as it is scattered under the skin near the spine and clavicle and can-not be isolated as a whole (169). White adipose tissue is responsible for storing energy such as lipid and is relatively abundant, whereas brown adipose tissue is a highly specialized type that consumes energy and generates heat in a mitochondrial uncoupling protein 1 (UPC1)-dependent manner, thus sustaining glucose homeostasis and enhancing insulin sensitivity. UPC1 is a mitochondrial carrier that mainly expressed in brown adipose tissue, facilitating heat generation by dissipating the protonmotive force rather than adenosine triphosphate (ATP). Beige adipose tissue also generates heat in a mitochondrial UPC1-dependent and independent manner (170–172).

Adipocytes can be activated into cancer-associated adipocytes(CAAs)in the presence of tumor cells, leading to the altered adipose function and paracrine signaling (167). CAAs play an essential role in promoting tumor progression by secreting multiple adipokines, such as CCL2, CCL5, leptin, and adiponectin (173). Besides, adipocytes can undergo metabolism reprogramming in the context of cancer. For instance, the secretions of BC cells can trigger the breakdown of adipocytes, releasing free fatty acids (FFA), which cancer cells can then utilize as an energy source to support their survival (174). However, adipocytes may also exert different roles depending on cancer types. Thus, it is important to elucidate the mechanisms through which adipocytes promote tumor development and select appropriate targeted therapies according to these mechanisms.

BC is a highly malignant cancer type characterized by its aggressive invasion, rapid proliferation and extensive dissemination. Adipocytes play an important role in the progression of BC (173). Adipokines, which are key secretions from adipocytes, promote BC progression. Leptin, as one of the most important adipokine, can promote the EMT of BC cells, thereby enhancing tumor metastasis, through the activation of the PI3K/AKT signaling pathway and the upregulation of pyruvate kinase M2 (PKM2) (175). Besides, adipocyte-derived leptin and IL-6 accelerate BC metastasis via Lysyl Hydroxylase-2 upregulation mediated by PI3K/AKT and JAK/STAT3 pathway (176). Adiponectin, another important adipokine secreted by adipocytes, has been shown to inhibit BC development. It is reported that adiponectin induces a robust autophagosomes accumulation, leading to BC cells apoptosis via STK11/LKB1-mediated activation of the AMPK-ULK1 axis (177). Moreover, adiponectin may inhibit BC development by reprogramming metabolism, suppressing fatty acid synthesis and stimulating lipophagy-mediated lipolysis fatty acid oxidation (FAO) (178). EVs are also important factors that are involved in tumor progression. CAA derived EVs have been shown to protect BC cells from drug-induced apoptosis *in vitro.* This protection may be associated with Hippo signaling pathway and blocking this pathway showed promise to suppress growth- promoting effect by CAA derived EVs (179). Adipocytes are also capable of secreting oleic acid, protecting TNBC cells from lipid peroxidation and ferroptosis when ACSL3 exists (180). Additionally, adipocytes also influence the function of immune cells in the BC microenvironment. Adipocytes-derived CXCL8 remodels the immune microenvironment of BC by suppressing the infiltration of CD4^+^/CD8^+^ T cells and upregulating CD274 (namely PD-L1) expression in TNBC (181).

Adipocytes can also regulate the progression of hematological neoplasms, such as acute lymphoblastic leukemia (ALL) and acute myeloid leukemia (AML) (182, 183). Marrow adipocytes (MATs) are crucial components of the bone marrow microenvironment and play an active role in promoting hematological neoplasms (182). AML blasts can hijack MATs metabolic procession and transfer fatty acids from the MATs to the AML blasts, providing energy to support tumor survival (182). As we elucidated before, adipocytes secret multiple adipokines to influence tumor progression. For instance, leptin, has been shown to contribute to hematological malignancies development via JAK/STAT pathway, which regulates the downstream signaling pathway such as PI3K/AKT signaling and ERK1/2 (184). Besides, high levels of leptin in concert with low adiponectin can increase the risk of blood cancer occurrence (185). However, MATs may also inhibit blood cancer growth. A study revealed that AML can suppress MATs, leading the dysregulation of endogenous hematopoietic stem and progenitor balance. The researchers subsequently administered a peroxisome proliferators-activated receptor (PPAR)γ agonist *in vivo* to induce adipocytes production and found that leukemia growth was repressed, suggesting that MATs may play dual roles in blood cancer development (186).

Adipocytes may also affect CRC progression through multiple mechanisms. CRC cells can reprogram adipocytes into CRC-associated adipocytes *in vivo*. They support cancer cell survival through secreting metabolites, such as lactate, and promote the lipid storage in cancer cells, thus providing energy to support their survival (187). Moreover, adipocytes are capable of secreting various adipokines. Adipocytes-derived leptin promotes colorectal carcinogenesis by binding to OB-R, a leptin specific receptor, causing a cascade of signals, including c-Jun, Akt, and JAK/STAT3 signaling pathways. Adiponectin, as another important adipokines, may serves as a potential anti-cancer factor that suppress CRC development via the activation of AMPK (188). Besides, adipocytes release TNF-α and IL-6 through Fas, NF-κB and MAPK signaling pathway. Increased TNF-α also promotes the lung metastasis of colon cancer (189). Adipocytes within the TME, may also contribute to the chemoresistance of CRC. Microsomal triglyceride transfer protein (MTTP) is especially increased in CRC patients with high fat ratio, this adipocyte-derived MMTP reduces the CRC patients’ reactivity to chemotherapy drug, oxaliplatin. The potential mechanism may be that the interaction between MMTP and proline-rich acidic protein 1 (PRAP1) reduces polyunsaturated fatty acids ratio and lipid reactive oxygen species (ROS) levels (190).

Adipocytes derived EVs can impact several critical traits of prostate cancer, such as elevated glucose consumption, lactate release and ATP production, thus promoting cancer proliferation, invasion and migration (191). This promoting effect may be associated with enhanced Akt/HIF-α axis-related Warburg effect (191). Besides, adipocytes directly stimulate the release of macrophage inhibitory cytokine-1 (MIC-1) from pancreatic cancer cells, which is associated with enhanced tumor progression, and IL-8 from prostate stromal fibroblasts through upregulating lipolysis and FFAs release (192). IL-8 recruits granulocytic/polymorphonuclear MDSCs (PMN-MDSCs) into the TME via IL-8/CXCR2 axis, continuously contributing to prostate cancer development (193). In addition, IL-8 hyperactivates PPARα to reduce glucose utilization and increase fatty acid catabolism. This effect significantly inhibits CD8^+^ T cell proliferation and weakens their anti-tumor effects (194). However, adipocytes may also serve as a potential protecter in the development of prostate cancer. Exogenous adiponectin protects normal tissues from the damage caused by radiotherapy. Meanwhile, it confers no protection for the prostate cancer cells (195). This suggests that adipocytes may also play a positive effect in prostate cancer. However, it remains unclear whether the administration of exogenous adiponectin interferes other signaling pathways in cancer cells, thereby promoting cancer progression. Therefore, more careful evaluations are needed to ensure the safety of exogenous adiponectin administration.

Adipocytes, traditionally regarded as energy storage cells, also serve as critical components in the TME. In the context of cancer, adipocytes supply energy, particularly in the form of FFAs to support tumor growth. Besides, adipokines secreted from adipocytes have been reported to be involved in multiple cancer progression. The study of adipocytes in the context of different cancer also provides us with more strategies to treat cancer based on adipocytes. It is promising to block the adipokines from adipocytes and disrupt adipocytes mediated metabolism. However, most of the drugs targeting adipocyte associated signaling still stay in the preclinical research. It is of great significance to transform the preclinical research into clinical practice.

### 2.5 Schwann cells

SCs, the most abundant glial cells in the peripheral nervous system (PNS) and indispensable components of the TME (196), play vital roles in nerve repair and regeneration. Schwann cell precursors (SCPs), originating from neural crest cells, first differentiate into immature SCs and later progress into different lineages: myelinating SCs and non-myelinating SCs (197). SCs are widely distributed throughout the body and they become an attractive target for cancer cells, particularly during the early stages of the tumor progression and TME formation (198, 199). In the early stage of tumor development, SCs can directly attract tumor cells, creating an injury-like microenvironment that facilitates perineural invasion (PNI) and tumor innervation (200). Through PNI, cancer cells spread to distant organs, leading to unfavorable dissemination. Additionally, SCs interact indirectly with other non-malignant cells, such as immune cells and CAFs in the TME, contributing to an immunosuppressive microenvironment (200). However, the role of SCs in the TME varies across different tumor types due to the tumor heterogeneity and understanding the cancer-promoting mechanisms of SCs in different cancers is crucial for cancer therapy.

Numerous studies have shown that SCs contribute to pancreatic cancer development through multiple mechanisms. SCs have been linked to delayed diagnosis of PDAC. PDAC-derived CXCL12 can induce the infiltration of SCs into the tumor site. By binding to CXCR4/CXCR7 on the SCs, SCs downregulate the expression of multiple pain associated targets to attenuate pain perception, while IL-6 secreted by activated SCs suppresses pain signaling, collectively delaying pancreatic cancer diagnosis (198, 201). Another example is that PDAC-derived tissue inhibitor of metalloproteinase 1 (TIMP1) stimulates CCL7 secretion from SCs and forms a paracrine feedback loop that continuously drives the invasion and metastasis of PDAC (202). Tumor-associated non-myelinating Schwann cell abundance was associated with an immunosuppressive environment and poor prognosis. These cells expressed plasmacytoma variant translocation 1 (PVT1) to promote the activation of kynurenine pathway in pancreatic cancer, contributing to tumor immune exclusion (203). Additionally, SCs secrete significant amounts of TGF-β, activating the TGF-β-SMAD signaling pathway in cancer cells, which is correlated with enhanced PDAC invasiveness (204). Moreover, another example that injecting cancer cells into sciatic nerve of nude mice to establish a dorsal root ganglion (DRG) co-culture system with cancer cell lines, found that pancreatic cancer cells induce SCs autophagy via NGF/ATG7 pathway, thus promoting pancreatic cancer PNI (205). Beyond these direct interactions, SCs engage with other cells in PDAC microenvironment. For instance, a study showed that SCs stimulate the proliferation and migration of PDAC cells through the Midkine signaling pathway and facilitate the transition of CAFs into iCAFs through the release of IL-1α (206). Previously, it has been elucidated that TGF-β signaling promotes the transformation of NFs into myCAFs instead of iCAFs by downregulating IL1R expression (98).

Several other studies have also demonstrated that SCs act as a potential promoter in melanoma. Melanoma cells can reprogram SCs into repair-like SCs (rSCs), initiating a nerve injury-like response. Once activated, rSCs significantly alter the melanoma microenvironment and promote tumor growth by modulating the immune system and remodeling the ECM both *in vitro* and *in vivo* (207). Moreover, SCs in the skin suppress anti-tumor response by downregulating pro-inflammatory signaling. Reprogrammed rSCs in melanoma increase the production of anti-inflammatory factors, such as prostaglandin E2 (PGE2), COX-2 and lipoxins A4/B4 to suppress anti-tumor T cells in a SCs-dependent manner (208). Furthermore, PGE produced by SCs also effectively inhibits proliferation of CD3/CD28 activated T cells and upregulates PD-1 expression on both CD4^+^ and CD8^+^ T cells (209). In addition to suppressing T cell proliferation, evidence also exhibited that SCs may inhibit dendritic cells (DCs) function. DCs co-cultured with melanoma-activated SCs can-not stimulate T cell activation and proliferation *in vitro*, suggesting that SCs may transform DCs into an immunosuppressive phenotype (210). Additionally, a recent finding indicated that dietary palmitic acid (PA) induced melanoma metastasis is associated with a pro-regenerative state of tumor-activated SCs (211).

Recent findings also reported that SCs may be involved in lung cancer progression. SCs can promote EMT and motility of lung cancer cells by elevating transcription factors Snail and Twist expressions. Blocking Snail and Twist expression significantly eliminated this enhanced motility. The potential mechanism is that SCs-derived CCL5 is responsible for lung cancer EMT. At the same time, CCL5 activates the PI3K/AKT/GSK-3 β/Snail-Twist pathway by binding to CXCR2, contributing to enhanced lung cancer invasiveness and dissemination (212). Additionally, SCs-derived exosomes containing miRNA-21-5p were proved to promote lung cancer proliferation and lymph nodes metastasis both *in vitro* and *in vivo*, suggesting that miRNA-21-5p may serve as a new therapeutic target for lung cancer (213). Furthermore, a previous study showed that stress-induced epinephrine elevation facilitates BC tumorigenesis by enhancing cancer stem cell (CSC) factors secretion (214). Besides, SCs contribute to lung cancer chemotherapy resistance by establishing an adrenergic microenvironment. Mechanistically, SCs express catecholamine-synthesizing enzymes and produce adrenaline, leading to enhanced chemoresistance of lung cancer via activating YAP/TAZ (215). In the meanwhile, SCs are reported to interact with immune cells in lung cancer, so that they help to establish an immunosuppressive microenvironment. SCs secret high levels of CCL2, promoting the M2 polarization of macrophages. In this study, CD14^+^ macrophages isolated from the co-culture system with SCs were used to treat A549 and H1299 lung cancer cells. It was observed that the proliferation of lung cancer cells increased. This finding suggests that SCs have the ability to interact with immune cells, thereby promoting the progression of lung cancer (216).

The PNS which has recently emerged as an important component of the TME, highlights SCs as key players in nerve-regulatory tumors. However, the studies of SCs are not as clear as other benign non-immune cells in the TME and there are no relevant specific strategies targeting SCs. However, the current studies offer us many enlightenments. IL-6 and CXCL12 have been reported to delay the early diagnose of pancreatic cancer in mice. But there are no relevant studies in human. It is promising to explore whether these two cytokines work in human, which would be beneficial to the diagnose of pancreatic cancer. Additionally, SCs also actively interact with immune cells, such as T cells and DCs, in the TME to form an immunosuppressive microenvironment. This could be also considered as a promising point to treat cancer. Targeting SCs not only holds promise for melanoma therapy but also provides a novel strategy for the early detection and treatment of pancreatic cancer.

### 2.6 Other cells

#### 2.5.1 Oligodendrocytes in glioblastoma

In addition to the benign non-immune cells mentioned above, there are also benign non-immune cells that participate in the development of certain cancer. For example, oligodendrocytes are important glial cells in the central nervous system and some studies have revealed that oligodendrocytes may support glioblastoma (GBM) development. The GBM border microenvironment has been reported to contribute to GBM’s chemo-radio resistance (217). They found an increase in oligodendrocyte progenitor cells (OPCs) and macrophages/microglia at the tumor border and observed enhanced stemness and chemo-radio resistance in GBM, suggesting that OPCs are critical components of this resistance (217, 218). Another study found that oligodendrocytes may promote GBM invasion through the Ang2 signaling pathway (219). Furthermore, GBM with oligodendrocyte components show a high mutation rate of IDH1 (220). A mathematical model further demonstrated that IDH1 mutated GBM shows a more invasive property compared to IDH1 wild type (221). However, oligodendrocytes may also be a promising target to treat GBM. A recent study revealed that GBM exhibit two different cell states in the core region and border, astrocyte-like cells and OPC-like cells, in the process of GBM invasion. Activator protein 1 (AP-1) exhibits high activity in the core region but low in the border, while BTB domain and CNC homolog1 (BACH1) shows the opposite. Combining AP-1 with BACH1 inhibitors produced a synergistic effect, significantly enhancing the tumor-suppressing capabilities compared to the use of single reagent (222). This study implicated that oligodendrocytes may be a promising target in the future study, which also offer us a new strategy to treat GBM.

#### 2.5.2 Mesenchymal stem cells in GBM

Mesenchymal stem cells (MSCs) are pluripotent stem cells with robust differentiation capacity. These cells are capable of differentiating into adipocytes, osteoblasts, myoblasts, *etc*. upon subjected to stimulation and serve as crucial components in maintaining tissue homeostasis and repair (223, 224). However, MSCs can also promote or suppress cancer progression through the secretion of cytokines, chemokines and exosomes (225). Recent studies exhibited that MSCs are critical components that are involved in GBM progression and the infiltration level of MSCs into GBM are always associated with poor prognosis (226). MSCs can promote GBM development through multiple mechanisms. For instance, mesenchymal stem like cells (MSLCs) enhance GBM invasiveness through the secretion of C5a, which activates p38 MAPK-ZEB1 signaling pathway (227). Tumor-associated mesenchymal stem cells (TMSCs) can induce the production of HAS2, which increases hyaluronan abundance in the TME, thereby promoting GBM invasiveness in a signaling ligand manner (228). Also, a co-culture experiment of MSCs and GBM cells proved that MSCs-derived secretions promote the activity, proliferation and migration of GBM cells (229). However, MSCs may also serve as a potential tumor-inhibiter in the TME of GBM. A recent study revealed that MSCs with high expression of CXCL10 and Nrf2 (an anti-apoptosis gene), remodeled the TME by recruiting CD8^+^ T lymphocytes (230). In recent years, studies have highlighted the crucial roles of MSCs, serving as a carrier loaded with anti-tumor drugs to treat GBM due to their strong tropism towards tumors. MSCs can be homed to the GBM site under the effect of multiple cytokines, such as TGF-β, CXCL12 (231, 232). MMP1 is also considered as an indispensable factor derived from MSCs that contributes to MSCs migration (233). According to this property of MSCs, many researchers try to develop MSCs loaded with different drugs, proteins, *etc*. to overcome GBM. For instance, a study constructed bone marrow derived-MSCs expressing miRNA-30c that effectively induce GBM cell apoptosis and impair tumor invasion (234). Another study designed more complicated MSCs that overexpress CXCL10 and Nrf2. These MSCs are capable of attracting CD8^+^ T cells to the GBM site and decreasing the levels of Tregs and exhausted CD8^+^/CD4^+^ T cells. Such recombinational MSCs have been demonstrated to significantly inhibit GBM growth (230). In conclusion, MSCs may serve as a potential drug carrier and in concert with T cells to overcome the challenges of tumor treatment.

#### 2.5.3 Epithelial cells in Esophageal Cancer

The role of epithelial cells in the progression of esophageal cancer has not been well studied, but some recent studies provided evidence that epithelial cells may participate in esophageal cancer development via several mechanisms. For example, the aberrant interaction of epithelial cells within the basal layer at early precancerous stage involves Ephrin-B1 (EFNB1) –Eph receptor B4 (EPHB4) which triggers EMT, leading to esophageal cancer tumorigenesis and progression (235). It is well studied that EMT can promote the invasion and metastasis of multiple cancers (236). Besides, epithelial cells promote esophageal cancer progression by activating fibroblasts into CAFs. Annexin1 (ANXA1), a ligand for the formyl peptide receptor type 2 (FPR2) on fibroblasts that can maintain fibroblasts stability, but gradually decreases as esophageal cancer progressed. The absence of ANXA1 leads to the uncontrolled transformation of fibroblasts into CAFs, and this transformation is enhanced by epithelial cells derived TGF-β (237). Epithelial cells may also be involved in tumor distant dissemination. In addition, a new subpopulation of epithelial cells have been identified in esophageal cancer, namely SAA1^+^ epithelial cells, that contribute to esophageal cancer distant metastasis by single cell profiling (238). Thus, targeting epithelial cells may also offer potential strategies to suppress esophageal cancer progression.

## 3 Role of immune cell types in the TME

Immune cells, which are primarily responsible for maintaining the immune balance of the body, paradoxically contribute to the creation of a tumor-promoting environment within TME (239). Immune cells within TME can be broadly categorized into two groups: lymphocytes and myeloid-derived immune cells. Both lymphatic cells and myeloid cells play critical roles as the drivers of tumor progression, demonstrating the dual and context-dependent functions of the immune system in cancer.

### 3.1 Myeloid-derived immune cells

Myeloid-derived immune cells are important immune cells in the TME, and are associated with tumor development. Myeloid-derived immune cells in the TME, including TAMs, MDSCs, *etc*., exert a dual role within the TME, typically exhibiting immunosuppressive effects (240). For example, TAMs in the TME secret multiple cytokines and proteins, such as epidermal growth factor (EGF), FGF, IL-10, VEGF, PDGF, *etc*., these factors not only promote tumor invasion and metastasis but also may contribute to the formation of tumor blood vessels, providing nutrition and energy for tumor growth (241–243). Moreover, TAMs recruit FOXP3^+^ Tregs, which exhibit immunosuppressive effects in the TME, in response to hypoxia, leading to the impaired T cell activation (244). MDSCs represent another major immunosuppressive population in the TME and can be classified into two subpopulations: PMN-MDSCs and monocytic MDSCs (M-MDSCs) based on their origin from the granulocytic or monocytic myeloid cell lineages (245). M-MDSCs are capable of producing high levels of NO to inhibit T cell proliferation and differentiation (246), while PMN-MDSCs exert their immunosuppressive effect through different mechanisms. These cells produce TGF-β, a potent immunosuppressive factor, to inhibit immune response (247). Besides, PMN-MDSCs suppress immune function in an antigen presenting manner and inhibit the production of ROS. The production of ROS also plays a crucial role in suppressing T cell immune response (248). Given the critical role of myeloid cells in TME, targeting these cells presents a promising therapeutic strategy for cancer treatment. Therapeutic strategies targeting CXCR2, C-C motif chemokine receptor (CCR)2, colony stimulating factor 1 receptor (CSF1R), PI3K-γ and STAT3 signaling pathways have also shown potential in inhibiting the immunosuppressive and inflammatory effects of myeloid-derived immune cells in cancers (249). In conclusion, myeloid cells in the TME are critical to tumor progression, so targeting myeloid cells in TME, especially those that play a powerful immunosuppressive role, provides a new strategy to cancer therapy.

### 3.2 Lymphatic cells

Lymphatic cells are also important components in the TME, including T cells, B cells and NK cells. T cells, upon activation by DCs and CD4^+^ T cell help signals, can differentiate into effector T cells (Teffs). Teffs are cytotoxic T cells that effectively eliminate target cells through mechanisms such as granule exocytosis, Fas/FasL interactions, and the secretion of cytokines like interferon-γ (IFN-γ) and TNF-α (250). Moreover, CD4^+^ cytotoxic T lymphocytes (CTL) may exhibit tumor-suppressing effect independent of CD8^+^ T cells. These CD4^+^ T cells showed tumor-suppressing effect on CRC growth in mice with humanized immune system by recognizing human leukocyte antigen class II (HLA-II) on tumors (251). NK cells are important immune cells that effectively eliminate cancer cells, especially blood cancers. However, these cells exhibit limited tumor-killing effects in solid tumors due to the complicated microenvironment that leads to NK cell inactivation and reduced tumor infiltration (252). Another important subtype of T cells, Tregs, displays immunosuppressive roles and can be attracted into the tumor site through the chemokine gradients, including CCL17/22, CCR8‐CCL1, CCR10‐CCL28 *etc*. Once there, they subsequently become activated and exhibit tumor-inhibiting functions (253). Tregs within the TME, can induce CD8^+^ T cell and NK cell death by releasing perforin and granzyme B, leading to impaired anti-tumoral immune response (254). Additionally, ATP-derived from Tregs inhibits the activation of T cells and antigen-presenting cells (APCs) (255). Immune checkpoints are proteins that regulate immune cell activity especially T cells, inhibiting their hyperactivation. Evidence showed that Tregs express co-stimulatory molecules including cytotoxic T lymphocyte-associated protein 4 (CTLA-4), PD-1, lymphocyte activation gene-3 (LAG-3), *etc*. to suppress immune response (256–258), and ICI are promising methods to reverse the immunosuppressive effect induced by Tregs. Additionally, reinvigorating exhausted CD8^+^ CTLs also holds promise for cancer therapy. Together, lymphocytes, particularly Tregs, play significantly immunosuppressive roles in the TME, and targeting these cells presents promising therapeutic options for enhancing cancer treatment.

## 4 Conclusion

TME is an intricately complex niche that has garnered significant attention in recent year. The TME of hematological malignancies differs substantially from that of solid tumors. While numerous promising strategies, such as chimeric antigen receptor T-cell (CAR-T) therapy, have demonstrated effective outcomes in treating hematological cancers, the treatment of solid tumors remains challenging due to their complex microenvironment and deep anatomical locations, which hinder drug infiltration into the tumor core. Additionally, the composition of the microenvironment varies across tumor types, presenting a significant challenge for cancer treatment. Recently, increasing attention has been directed towards benign non-immune cells within the TME, which may offer novel avenues for cancer therapy.

CAFs are abundant and heterogenous benign non-immune cells comprising diverse subtypes in the TME that have been extensively studied. CAFs in the TME secret multiple tumor-promoting cytokines, such as IL-6 and TGF-β to support tumor growth. Additionally, CAFs contribute to ECM remodeling, which restricts drug infiltration and promotes cancer dissemination. Targeting CAFs may represent a promising strategy to inhibit tumor development. FAP are proteins that highly expressed in CAFs across multiple cancers. Targeting FAP to eliminate CAFs combined with chemotherapy drugs maybe an effective way to inhibit tumor growth. A study showed that FAP-targeted vaccine effectively enhanced the CAFs-killing effect mediated by CD8^+^ T cells and increased drug uptake by colon cancer and BC (259). TGF-β is a critical factor that actively participates in tumor progression, such as immunosuppressive effect and ECM remodeling. Blocking TGF-β holds promise for new cancer therapies. CTL are often suppressed by elevated immune checkpoints and can-not effectively infiltrate into the tumor core due to the physical barrier. Inhibiting TGF-β combined with immunotherapies to enhance T cells infiltration also holds promise. M7824, a fusion protein against PD-L1 and TGF-β extended overall survival and provided long term protective effects (260). Additionally, some CAFs derived cytokines also participate in tumor development. Blocking these cytokines also sheds light on cancer therapy. IL-6 has been reported to promote cancer progression through multiple pathways. Antibodies blocking IL-6 combined with PD-1 were proved to strikingly elevate survival rate of mice with pancreatic cancer (95). However, there are also potential limitations behind these therapies. CAFs exhibit high heterogeneity and could be divided into multiple subtypes with different markers. Some normal tissues have low levels of FAP expression, FAP-targeted methods effectively eliminate CAFs as well as hurting normal tissues or organs. A study revealed that the depletion of FAP^+^ stromal cells leads to the cachexia and anemia (261). Besides, not all CAFs express FAP so that targeting FAP^+^ CAFs to reverse the immunosuppressive effect is not realistic (262). Single FAP-targeted therapy does not achieve good outcomes, so it is better to be used in combination with ICI. At the same time, FAP-targeted therapy does not guarantee the elimination of CAFs that are detrimental to tumor progression, and it may also eliminate CAFs that inhibit tumor growth. For example, a study revealed that the depletion of myCAFs increased CD4^+^Foxp3^+^ Tregs infiltration into tumor, leading to an aggressive tumor progression (263). Blocking TGF-β or degrading ECM represents another method to promote drug infiltration into cancers, such as pancreatic cancer which are surrounded by dense ECM barrier, thus contributing to cancer therapy. The degradation of ECM may increase the risk of tumor metastasis, so it is important to apply these methods based on tumor types. Appropriate ECM allows drug infiltration and reduced tumor metastasis. To overcome these obstacles, it is pivotal to develop new therapies or improve the existing methods. Considering that the direct elimination of CAFs may lead to unintended tumor-promoting effect, the transformation of tumor-promoting CAFs into tumor-suppressing CAFs may shed light on tumor therapy. An advanced technology, termed hydrogen therapy, was proved to reverse the immunosuppressive and tumor-promoting phenotype of CAFs, thus remodeling the immunosuppressive TME. This therapy directly kills cancer cells, suppresses immunosuppressive factor derived from CAFs and stimulates systematic immune response (264). As for FAP-targeted therapy, enhancing the target specificity and in concert with other therapies may provide better treatment. A novel FAP-based method, molecular pro-theranostic probe (FMP) with activatable fluorescence, photoacoustic (PA) imaging, and photodynamic therapy (PDT), is able to completely repress primary tumor. And the tumor-killing effect could be further enhanced in combination with ICI (265). The development of nano-drugs, also offers us some enlightenments. These drugs play a role by interfering the immunosuppressive effect mediated by CAFs, eliminating CAFs and inhibiting CAFs activation (266).

ECs within the TME often promote the formation of tumor neovascularization, supplying energy to promote tumor growth and metastasis. Targeting ECs to suppress blood vessel formation in tumor mass may be an effective adjuvant therapy. Bevacizumab was approved to treat metastatic CRC by specifically blocking VEGF (267). Sunitinib and sorafenib can inhibit VEGF receptor tyrosine kinase, thus suppressing tumor blood vessel formation (268, 269). Besides, targeting Ang-Tie2 axis is also a promising method. Dual inhibition of VEGFR and Ang2 was proved to normalize tumor blood vessel and prolong overall survival (270). This experiment has demonstrated the therapeutic effects of anti-Ang2 drugs in mice, but remains unclear in humans. There are also potential risks behind anti-angiogenic therapy. Drug resistance often emerges in this therapy, this is partly due to the complicated angiogenic mechanism of cancers. Since drugs target the relevant signaling pathways to inhibit tumor vascular formation, tumor cells rapidly evolve to upregulate other pro-angiogenic factors, such as FGF, PDGF, and switch to other pro-angiogenic pathways, resulting in drug resistance (143, 144). Furthermore, the destruction of tumor vascular system may lead to hypoxic microenvironment that contributes to an enhanced drug resistance and angiogenic capacity of cancer cells (271, 272). Therefore, it is better to develop combinational therapies. Drugs that target multiple angiogenic signaling pathways or anti-angiogenic drugs in concert with ICI may achieve better outcomes. Additionally, a recent study revealed that fructose transporter SLC2A5 is significantly upregulated in ECs of HCC, this alteration facilitates the angiogenesis of HCC especially under hypoxia (122). It suggests that targeting EC associated metabolic pathway may be also a promising strategy. However, whether this target plays a role on human needs further exploration.

PCs can be considered as the gatekeeper of the blood vessel, playing a significant role in sustaining vascular stability. PCs are also actively involved in tumor vascular formation and stability. However, most anti-angiogenic strategies predominantly focused on ECs. Targeting PCs associated markers offers additional methods to suppress tumor angiogenesis. Sustaining PDGFR-β in a non-active state showed significant anti-angiogenic and tumor-suppressing effect in mice with pancreatic cancer and renal carcinoma (273). Imatinib effectively reduced vascular density and increased blood vessel permeability, thus inhibiting lymphoma growth (274). This method is based on targeting PDGFR-β^+^ PCs. However, targeting PCs faces the common problems of anti-angiogenic drugs. Drug resistance and drug-induced hypoxia in the TME make drug combination necessary. Combining PCs-targeted drugs with chemotherapy drugs or ICI may achieve better outcome compared to single drug. As for novel therapies, a α-polarized DC vaccine targeting PC antigen DLK1, protected mice from CRC progression by enhancing CD8^+^ T Cell mediated anti-tumor immunity (275). In the future, it is possible to identify specific markers of PCs and develop novel combinational drug therapies.

Adipocytes are energy store and endocrine cells that provide energy to support tumor growth, secret factors to suppress immune cell function and transform them into immunosuppressive phenotypes. There are already many strategies to reduce the pro-tumor effect induced by adipocytes. Such as blocking adipocytes derived factors, disrupting the energy source of tumor cells and reversing the immunosuppressive microenvironment. Leptin has been proved to promote the progression of multiple cancers, such as BC, blood cancer and CRC. Blocking leptin is promising to reduce adipocytes mediated tumor-promoting effect. Celastrol, a substrate that binds to leptin receptor, significantly inhibits BC proliferation and migration by suppressing leptin mediated PI3K/AKT signaling pathway (276). FAO is known for fueling tumor cells and support their survival. Etomoxir can interrupt the progression of FAO and acquired better outcome when combined with temozolomide to combat GBM (277). However, there are also many obstacles need to be overcame. Tumor cells are surrounded by dense ECM, which makes it difficult for drug to infiltrate. Off-target effects and drug resistance are also worth considering. Tumor cells may upregulate other signaling pathways when certain signal is blocked. In recent years, there are also some novel strategies to cure cancer based on adipocytes, which would enlighten us. Adipocytes can be engineered to specifically deliver drugs to tumor cells. Adipocytes loaded with anti-cancer fatty acid, rumenic acid, and a doxorubicin prodrug can specifically deliver drugs to the tumor via lipolysis (278). According to this property, adipocytes may also serve as a potential drug carrier to delivery diverse drugs to tumor to overcome the physical barrier. A recent study developed a ground-breaking strategy, they designed engineered adipocytes which significantly compete for the energy and nutrition required for tumor cell survival. This method has been shown to strikingly reduce tumor growth, angiogenesis and hypoxia in mice with pancreatic cancer or BC (279). But whether this method works in human remains unclear. A recent study also revealed that a ketogenic diet in concert with eFT508, a P-eIF4E inhibitor, effectively inhibit pancreatic cancer growth by blocking fat metabolism that supplies energy for cancer cells (280). Therefore, blocking blood vessels derived energy, such as muti-target anti-angiogenic drugs, combined with fat metabolism inhibitors may be a promising method to inhibit tumor growth. However, further studies are needed to prove its feasibility.

SCs, largely unexplored benign non-immune cells in the TME, may not serve as a negative bystander but an active promoter in tumor progression, especially nerve-rich organs, such as pancreatic cancer. Most SCs-targeted strategies remain in the pre-clinical phase. It is unclear how much SCs contribute to tumor progression compared to other benign non-immune cells in the TME. SCs are able to secret multiple pro-tumor factors, such as IL-6, CXCL12, PGE2, *etc*. to participate in tumor progression. IL-6 and CXCL12 have been reported to delay the early diagnose of pancreatic cancer. However, no clinical studies have shown that these two cytokines also cause the delay of diagnosis in human with pancreatic cancer. In the meanwhile, SCs are capable of interacting with other non-malignant cells in the TME, such as DCs, TAMs and CAFs, to form an immunosuppressive microenvironment. Such microenvironment could be reversed to a certain extent under the effect of ICI. In the future, it is important to develop SCs-specific targeted therapy. YAP is a key molecule that promotes the proliferation and remyelinating of SCs via activating Hippo pathway in patients with neurofibromatosis type 1 (NF1) and SCs are characterized by high levels expression of YAP in NF1 (281). Therefore, YAP maybe a promising target to achieve targeted therapy based on SCs.

**Figure 2:**
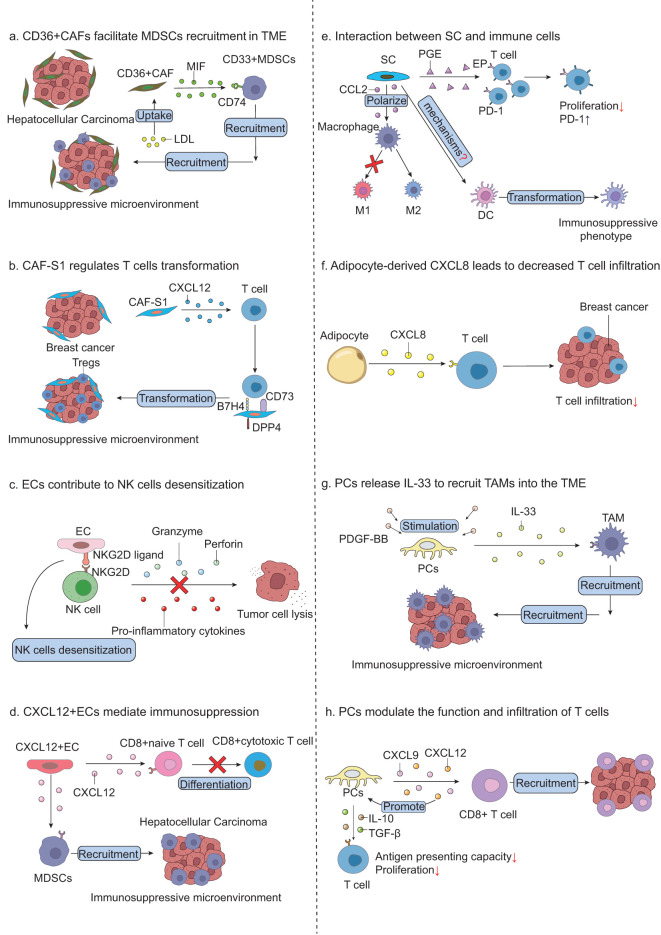
Crosstalk between benign non-immune cells and immune cells. **(a)**, CD36^+^ CAFs facilitate the uptake of LDL, thereby inducing MIF secretion and recruiting CD33^+^ MDSCs into the HCC microenvironment by binding to CD74 receptor on CD33^+^ MDSCs. **(b)** CAF-S1 attracts T cells by secreting CXCL12 and transforms T cells into Tregs via CD73, B7H4 and DPP4, leading to an immunosuppressive microenvironment in BC. **(c)** ECs desensitize NK cells through expressing NKG2D ligands, blocking NK cells-dependent tumor-killing effects. **(d)** CXCL12^+^ ECs block the differentiation of CD8^+^ naive T cells into CD8^+^ cytotoxic T cells by secreting CXCL12, as well as recruiting MDSCs into the TME of HCC by binding to the receptors on MDSCs. **(e)**, SCs secret PGE to inhibit T cell proliferation and upregulate PD-1 expression on T cells. Besides, SCs-derived CCL2 polarizes macrophages into M2- like phenotype, which is associated with reduced proinflammatory capacity. SCs co-cultured with DCs can transform DCs into immunosuppressive phenotype, weakening the antigen-presenting capacity of DCs. **(f)**, Adipocytes-derived CXCL8 reduces T cell infiltration into BC microenvironment. **(g)**, Under the stimulation of PDGF-BB, PCs secret IL-33 to recruit TAMs into the TME and form an immunosuppressive microenvironment. **(h)**, PCs secret CXCL9 and CXCL12 to recruit CD8^+^ T cells into the TME. CXCL12, in turn, promotes the release of TGF-β and IL-10, which weaken the proliferation and antigen-presenting capacity of T cells.

In conclusion, understanding the complex and dynamic nature of the TME and the specific mechanisms by which benign non-immune cells contribute to tumor progression remains a considerable challenge. This review highlights the multifaceted roles of benign non-immune cells in the TME but does not exhaustively elucidate their underlying mechanisms due to the complexity of this system (**Figure 2**). Future research should focus on tumor heterogeneity and the functional roles of benign non-immune cells in various cancers. In order to provide more cancer therapies, it is better to find out more specific markers on benign non-immune cells, which allows more accurate targeted therapy. Additionally, novel drug delivery systems offer us opportunities to orient cancer cells accurately, such as nanoparticle drugs, overcoming the difficulties of drug infiltration. At the same time, the combination of drugs significantly improves the therapeutic effects, reduces drug resistance and tumor compensatory effect. Patients will benefit from more effective combinational methods used in the clinical practice. This review has elucidated many promising targets on benign non-immune cell, but most research still stay in the preclinical stage, translating these findings into clinical practice would hold great promise for enhancing the overall efficacy of cancer treatment.
